# Investigation of Enhanced Ambient Contrast Ratio in Novel Micro/Mini-LED Displays

**DOI:** 10.3390/nano11123304

**Published:** 2021-12-06

**Authors:** Ke Zhang, Tingting Han, Wai-Keung Cho, Hoi-Sing Kwok, Zhaojun Liu

**Affiliations:** 1 Department of Electrical and Electronic Engineering, Southern University of Science and Technology, Shenzhen 518055, China; kzhangao@connect.ust.hk; 2 State Key Laboratory of Advanced Displays and Optoelectronics Technologies, The Hong Kong University of Science and Technology, Hong Kong 999077, China; eeterencecho@ust.hk (W.-K.C.); eekwok@ust.hk (H.-S.K.); 3 Shenzhen Refond Optoelectronics Co., Ltd., Shenzhen 518055, China; aidit.han@refond.com

**Keywords:** mini-LED and micro-LED display, emissive display, ambient contrast ratio

## Abstract

In recent years, ambient contrast ratio (ACR) has become very critical for advanced outdoor displays, including transparent displays, portable displays, and so on. In this work, the ACR of typical flat panel displays was introduced, while LED-based displays showed distinctive advantages. Micro-LED displays with a different pitch of 10 μm, 15 μm, 30 μm, and 60 μm were fabricated and characterized. Various mini-LED and micro-LED panels were systematically investigated in the aspect of brightness, reflection phenomenon, and ACR to reveal their enormous potential for outdoor applications. Through a series of experiments and comparisons, three methods were proposed to further improve the ACR of LED-based panels, including optical method, antireflection coating, and structure optimization.

## 1. Introduction

Recently, LED-based displays have attracted tremendous attention in many fields because of their superior properties such as self-emission, high brightness and efficiency, low power consumption, long lifetime, and wide operating environment [1,2,3,4]. They are regarded as promising candidates for next generation display, including some advanced display applications such as head mounted display (HMD), head-up display (HUD), wearable displays such as smart watches, AR/VR glasses, and so on [5,6,7,8,9,10]. Considerable efforts have been carried out by industry and academic institutes with fast-evolving technologies and prototypes.

The LED-based displays can be classified into three categories: fine-pitch LED displays, mini-LED displays, and micro-LED displays. Fine-pitch LEDs are mostly used for backlight units (BLU) with a new concept of local dimming, in which the backlight is divided into several parts and selectively driven. Mini-LED and micro-LED are applicable in emissive displays. For mini-LED display, large screen products are the mainstream, while transparent, portable applications are also very promising in the future [11,12]. The mini-LED display has a large application area and has different requirements. For outdoor display or cinema screens, high brightness is very important. While automotive screens and portable displays such as a smart watch require low power consumption and high reliability. The innovative technologies and products of high pixel per inch (PPI) micro-LED display were also witnessed and had a notable impact on the micro-display field [13,14,15,16]. The most attractive application is AR/VR, which requires high ACR, reliability, and low power consumption. As self-emissive devices, mini-LED and micro-LED can provide higher brightness, contrast ratio and efficiency, which are more suitable for low power consumption applications.

**Figure 1:**
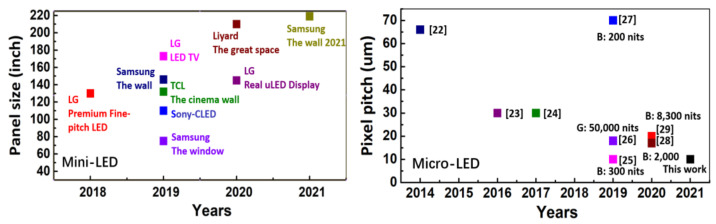
Development of mini-LED displays and micro-LED displays [22,23,24,25,26,27,28,29].

In recent years, displays do not restrict to the form of a flat panel. Many future applications, such as immersive stage shows, AR glasses, and smart watches, all require good outdoor display quality and stability [17]. Ambient contrast ratio (ACR), which means the contrast ratio in the presence of ambient light, becomes a significant factor for emissive display [18]. ACR has already been widely applied to estimate the sunlight readability of transflective liquid crystal displays (LCDs). However, the results are usually not good enough because of the low light efficiency of LCD-based products [18]. Then this concept is also extended to other flat panel displays (FPDs) such as OLED. However, OLED is not suitable for outdoor applications because of the relatively lower efficiency and faster degradation under high-current density, which may result in insufficient brightness [19]. Moreover, the instability of organic material makes it undesirable to be exposed to the ambient environment such as high temperature or high humidity conditions [20]. Mini-LED and micro-LED displays, with the significant advantages of high brightness and good stability, are expected to be superior for outdoor applications [21]. Figure 1 show the development of mini-LED and micro-LED displays. It is obvious that the panel screen of the mini-LED display is gradually increased, while the pixel pitch of the micro-LED display tends to be reduced [22,23,24,25,26,27,28,29].

In this paper, the fabrication and characterization of micro-LED displays with a pixel pitch of 10 μm, 15 μm, 30 μm, and 60 μm, were reported. Micro-LED prototypes were finally achieved through monolithic integration, with a brightness of 40,000 nits for blue and 120,000 nits for green. The total power consumption was about 0.94 W. Compared with other good prototypes in recent years [26,27,29], we have higher brightness and lower power consumption owing to the optimized fabrication process of micro-LEDs with a sapphire substrate. The far-field luminance distribution and full width at half maxima (FWHM) are comparable and even better with others. In the future, we will also focus on the light extraction efficiency to further improve the quality of the prototypes.

The principle of ACR was illustrated to form the specific model for LED-based display. Micro-LED and mini-LED (provided by Refond Optics) panels were systemically analyzed and compared to evaluate the outdoor performance. Three methods, including optical method, anti-reflection coating and structure optimization, were proposed to improve the ACR of LED-based display.

## 2. Fabrication and Characterization

The epitaxy grew on a 4′′ sapphire substrate by metal-organic chemical vapor deposition (MOCVD) from commercial LED producers. The epitaxy wafer includes a thin p-GaN layer about 150 nm doped by Mg, ten pairs of InGaN/GaN, a multiple quantum wells (MQW) layer, and a 5 μm thick n-GaN layer. To start with, the epi wafer was cleaned using aqua regia, H_2_SO_4_ + H_2_O_2_ and MS200l solution, in sequence to remove the potential inorganic and organic particles and thin oxidation layer. Meanwhile, to form micro-LED devices and arrays, the epi wafers were dry-etched to insulate pixels by ICP GaN-etcher with SiH_4_ and N_2_O in a high-power process of 20 W to clean the byproducts. Subsequently, the current spreading layer (CSL) was evaporated with Ni/Au of 40/40 A thick using negative photoresist and lift-off process. Following the annealing process performed under an atmosphere of N_2_:O_2_ = 4:1 and temperature of 570 °C for 5 min, both the p-electrode and n-electrode (ELEC) were formed simultaneously by evaporating metal stacks of Ti/Al/Ni/Au with 200/1500/500/800 A thick. In this work, a common n-electrode was adopted to reduce the pad numbers and increase the pixel density. In addition, the individual p-electrode design was used, which can also work as a mirror to reflect the light to the environment so that the light extraction efficiency could be increased. Additionally, more surrounding n-pads were applied to achieve a higher connection yield. N-metal gridding was deposited in the array on the top of the n-GaN area to improve the electrical uniformity and optical quality of the array [30]. A dielectric layer of 60 m SiN_2_ as a passivation layer was deposited at 300 °C by plasma-enhanced chemical vapor deposition (PECVD), on which a contact hole was opened. At last, indium was applied as a bonding pad by thermal evaporation. After the reflow process, a micro-LED with an indium solder bump was obtained.

**Figure 2:**
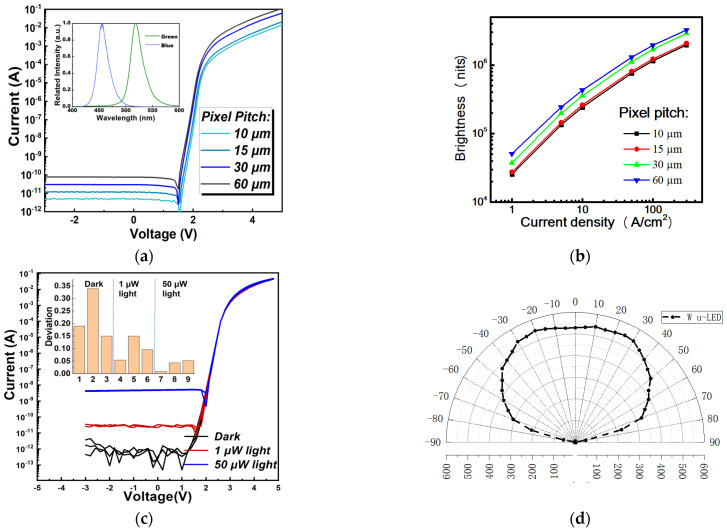
(**a**) I–V characteristics of micro-LED devices with pixel pitch of 10 μm, 15 μm, 30 μm and 60 μm. (**b**) optical brightness versus pixel size under different injected current density. (**c**) The ambient light effect on the devices with different illumination intensities. (**d**) Far-field luminance distribution of micro-LED displays.

The I-V characteristics of micro-LEDs are shown in Figure 2a. The forward voltage is approximately 2.7 V, and the leakage current is less than 100 pA. The optical characteristic of brightness versus pixel size under different injected current density are shown in Figure 2b, in which the light output power has a positive correlation relationship with the injected current. The brightness per area almost remains the same as the changed pixel size. The electroluminescence spectrum (EL) was shown in the inset of Figure 2a. The peak wavelengths of green and blue panels were 545 nm and 455 nm with an FWHM of 23 nm and 21 nm, respectively.

Ambient light is a critical factor for ACR research. Therefore, the relationship between the light illumination and device performance was also investigated, as shown in Figure 2c. It is clear that the leakage current (I_L_) increased gradually with the rise of light intensity. The I_L_ is 1 pA under dark conditions, 20 nA under 1 μW light illumination, and 7 nA under 50 μW light illumination. While the forward current (I_F_) also increased because of the photo-induced electron-hole pairs. In this case, the on–off ratio of micro-LED devices decreased dramatically. However, the I_L_ under strong illuminance is still too negligible to function as lighting or display applications. Devices across the 4′′ wafers for each condition were compared to show the small data deviation as shown in the inset of Figure 2c. Besides, ambient light also affects slightly on the slope [31]. According to the results, the light generated phenomenon is irrelevant to device size.

The far-field radiation distribution of the micro-LED micro-displays was measured and shown in Figure 2d to analyze the optical uniformity from different viewing angles. The micro-display demo was rotated from −90 degrees to 90 degrees. From the results, the micro-display panel has good optical uniformity and symmetry, with a large view angle of about 140° from −70° to 70°.

## 3. ACR Principle

**Figure 3:**
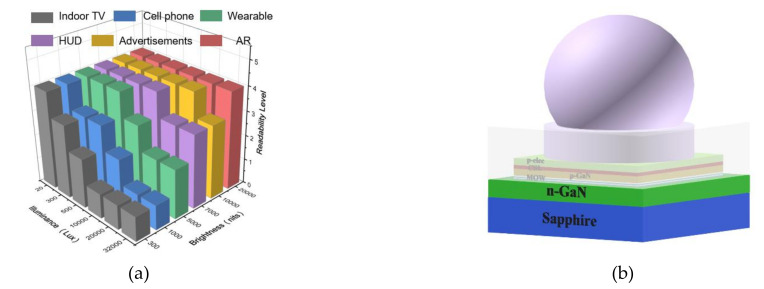
(**a**) ACR level comparison of potential outdoor products under different operation situations. (**b**) Three-dimensional structure of a micro-LED unit.

ACR means the contrast ratio in the presence of ambient light, which is often used in our daily life among many display products, as shown in Figure 3a. For example, current indoor TV, with usually hundreds of nits, only have good readability when ambient illuminance is smaller than 500 lux. Thus, its application would be limited in the room. For current cell phones or pads, the highest brightness is about 1000 lux. Products in this range could maintain readability in the room or under the outdoor shadow. However, the ACR would become unacceptable when it is exposed to strong sunlight. For LED-based panels with a brightness of over 7000 nits they can have adequate readability outdoor under a clear sky. If the brightness could further increase to nits, it would still maintain good contrast even under strong direct sunlight. Accordingly, brightness is one of the most important factors for ACR.

As a key metric of display applications, ACR usually becomes a serious limiting factor for many FPDs [32]. For example, the ACR of LCD (a non-emissive display) is very poor, caused by low light efficiency, because of the existence of polarizer and color filter. For OLED, large electrodes are usually used to increase the current uniformity as well as the light extraction efficiency. However, the metal electrodes may also reflect the ambient light, leading to the reduction of ACR and display view quality.

Min-LED and micro-LED displays also suffered a similar question. The device structure is shown in Figure 3b. Stacks of different metals are used as electrodes (ELEC). A larger ELEC area is desirable, which can achieve better electrical uniformity and avoid the current crowding phenomenon, which has been recorded in previous research results [30]. In this way, light is emitted from the MQW to the top and through the sapphire to the environment. Meanwhile, it can also be reflected by the electrodes to increase the light extraction efficiency $\eta_{LE}$, which correspondingly influences the external quantum efficiency (EQE) from Equation (1). Here, $\eta_{IJ}$ is the injection efficiency, and $\eta_{IQ}$ is the internal quantum efficiency.

(1) $$\eta_{EQE}=\eta_{IJ}\times \eta_{IQ}\times \eta_{LE}$$

Although ELEC could effectively increase the $\eta_{LE}$, LED-based displays may also suffer the *ACR* problems according to the contrast ratio defined as Equation (2). The display brightness on the off-state (*L_off_*) will also increase as the ELEC area. To specifically evaluate the performance of panels under ambient conditions for outdoor applications, the factor of *ACR* was defined as Equation (3) [18].

(2) $$CR=\frac{L_{on}}{L_{off}}$$

(3) $$ACR=\frac{L_{on}+L_{ambient}\times R_{L}}{L_{off}+L_{ambient}\times R_{L}}$$

Here, one of the most important factors to estimate *ACR* is the *R_L_*, the reflectance ratio. It can be calculated by equations [18], with the reflection spectrum and ambient light spectrum, and can also be measured with a real light source and the radiation detector. In the following experiment, the relationship between reflection and display brightness was specifically investigated, showing how the spontaneous properties make LED-based products almost the best candidate in outdoor display applications [33,34,35].

## 4. ACR: Experiment and Measurement

Although LED-based products have enormous advantages compared with other FPDs, it is still necessary to further improve the ACR. High brightness means high power consumption, which is very undesirable for wearable devices. Working with high brightness for a long time may also reduce the lifetime of the devices and panels. Here, we proposed three methods to improve the ACR of LED-based emissive displays.

### 4.1. Optical Method

**Figure 4:**
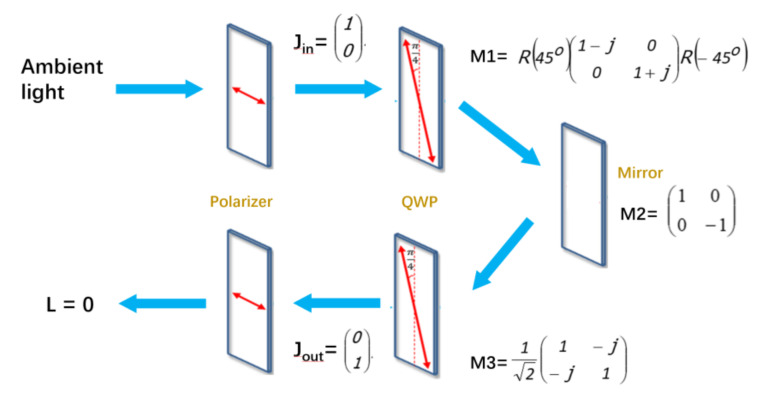
The optical system to eliminate ambient light reflection, including the jones matrix calculation to show the principle.

An optical method was proposed here, with some optics such as a linear polarizer and a quarter-wave plate (QWP). As shown in Figure 4, ambient light will be polarized as linear x-direction after passing through the first polarizer. The light was represented by a matrix of J_in_, which will be changed to RCP 9 right circular polarization by the QWP at a 45° axis to the polarizer with the matrix of M1. A following metal layer, ELEC, with a matrix of M2, tuned the light to left circular polarization (LCP). After passing through the QWP again with a matrix of M3 (the axis is mirrored), a linear y polarization light was obtained, which is J_out_. If the polarization direction is perpendicular to the original linear polarizer, J_out_ will be blocked. In this way, the reflection phenomenon can be almost eliminated, while the emitting brightness may be sacrificed by the optical system as well. The Jones matrix was calculated to ensure the elimination of the ambient light, as shown in Figure 4. [36].

The detailed reflection and brightness were measured with an area light source. After passing through the optical system, including a polarizer and QWP, *L_off_* reduced owing to the elimination of reflection. Therefore, ACR was raised to eight times. In detail, ACR increased from about 2861:1 and 436:1 to:1 and 3015:1, respectively, for mini-LED and micro-LED displays. The brightness (*L_on_*) is very close, being 6500 nits and 4900 nits. The reflection of micro-LED panels is roughly four times larger than that of the mini-LED panel because of the difference in the pixel proportion (PP). The PPs for micro- and mini-LEDs are 28% and 75%, respectively. Larger pp means larger ECR, leading to more serious ambient reflection problems. As a result, the final ACR of the micro-LED panel is approximately seven times compared to that of the mini-LED panel.

### 4.2. Antireflection Coating

**Figure 5:**
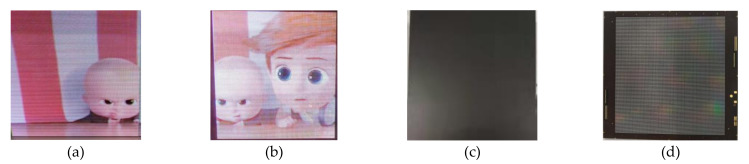
Mini-LED panels with and without coating under (**a**,**b**) on state and (**c**,**d**) off state.

The second method is to use an anti-reflection coating. Black packaging glue was a coating on the top of the panel [37], as shown in Figure 5. Three types of mini-LED panels, provided by Refond Optics, were investigated. They consist of blue pixels with the peak wavelength of 460 nm, RGB pixels and RGB pixels with an anti-reflection coating, respectively. The pixel pitch of panels is 700 μm with a resolution of 120 (×3) × 128. They are driven by a printed circuit board (PCB). Taking the RGB panels as an example, it is clear the panel with anti-reflection coating has better color purity because the interference of ambient light is eliminated. Under the off state, the panel without coating shows obvious light reflection and even halos, as shown in Figure 5.

### 4.3. Structure Optimization

**Figure 6:**
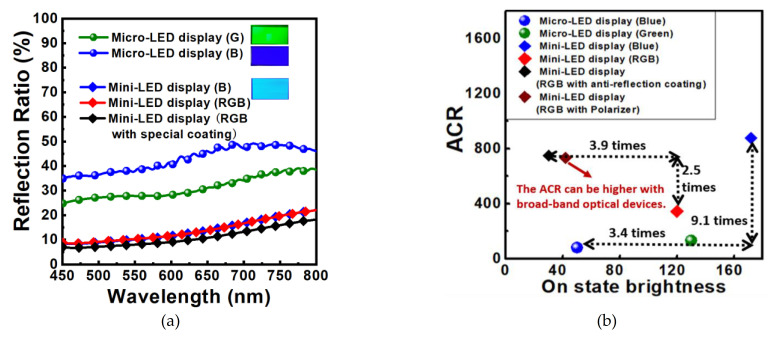
(**a**) reflection spectrum and (**b**) The ambient contras ratio versus on state brightness (knits) of different mini-LED and micro-LED panels.

Three types of mini-LED panels and two types of micro-LED panels with a peak wavelength of 460 nm and 520 nm were prepared. The reflection ratio of LED-based panels with different pixel pitches and pixel sizes were measured and analyzed, as shown in Figure 6a. Inset figures are the blue and green micro-LED panels fabricated by ourselves and blue mini-LED panels provided by Refond. Optics. It is noted that blue panels have a higher reflection ratio due to the low absorption from the material. The reflection of mini-LEDs is significantly lower than micro-LEDs owing to the low electrode cover ratio, revealing that the pixel size and the pixel pitch have an obvious influence on ACR. The large screen may not suffer serious reflection problems. For example, Sony reported a micro-LED screen in 2016 and claimed only 1%-pixel occupation, the electrode cover ratio even less. In this case, the ambient light reflection phenomenon is negligible [38].

Surface treatment can improve the ACR as well, which can be observed by comparing the micro-LED arrays on a flat sapphire substrate (FSS) and patterned sapphire substrate (PSS). Four micro-LED arrays with the pixel pitch of 60 μm, 30 μm, 15 μm and 10 μm, respectively, were fabricated and measured. The corresponding ECRs are 60%, 45%, 38%, and 25%, showing different ambient reflection ratios. It can be concluded that higher ECR leads to stronger reflection. The sample based on blue epis has higher reflection under the short wavelength light. Moreover, micro-LED arrays on PSS have much lower reflection compared with that of FSS, which is valuable to further investigate the surface treatment in the future.

In summary, the reduction of ECR will increase the final ambient contrast ratio, with the reduction of the electrical efficiency [39]. There is a trade-off between the reflection ratio and the electrical performance. High brightness of LED-based products can get better with ACR compared to other FPDs of the same power consumption; therefore, it is possible that some brightness can be sacrificed to improve ACR.

### 4.4. Discussions

The on state and off state radiation of the panels were tested, illuminated by three kinds of area light sources, with the color blue, white, and yellow, respectively. The detailed measurement of *L_on_*, *L_off_*, and ACR under those three conditions are shown in Table 1. The data under white illumination was further investigated to compare the difference of ACR and the sacrifice of the brightness illustrated in the characteristics of brightness versus ACR in Figure 6b. The ACR of the blue mini-LED panel is 8.1 times higher than that of the blue micro-LED panel. With the coating, the brightness of RGB mini-LEDs will be reduced by 3.9 times, while the ACR will still be increased by 2.5 times. Therefore, the AR coating method is a little better to get higher ACR. Besides, the AR coating method is easier and lighter compared with a complicated optical system. LED-based products are usually very stable and reliable, with no need for complicated protection material or designs to protect; therefore, AR coating can also be used as the package layer.

## 5. Conclusions

In this work, typical flat panel displays were introduced, while LED-based displays showed distinctive advantages for outdoor applications. Various micro-LED displays and mini-LED displays were analyzed and compared, including both monochrome and RGB panels. In conclusion, the reflection of mini-LEDs is significantly lower than Micro-LEDs owing to the lower pixel occupation. Therefore, high brightness is the most critical factor for achieving a high-quality display. However, electrode design and composition have a big influence on the micro-LED, for which special external methods were proposed to get enough ACR. To improve the ACR of the LED-based panel, three methods were proposed involving the optical method, anti-reflection coating, and structure optimization. The AR coating method showed better performance under different ambient light illumination conditions because of higher ACR, lower brightness scarification, and a light and simple design. To compensate for the trade-off between ACR and electrical efficiency, better ohmic contact, high current uniformity, and high efficiency were required. Therefore, structure optimizations were reported to analyze the size, pixel proportion, and ECR effect, which can be further investigated in the future.

## Figures and Tables

**Table 1:** Optical measurement of mini-LED and micro-LED panels illuminated by area light source with different reflection treatments.

Display Panel (uW)	Micro-LED Panel (B)			Micro-LED Panel (G)			Mini-LED Panel (B)			Mini-LED Panel (RGB)			Mini-LED Panel (RGB with Coating)			Mini-LED Panel (RGB with Polarizer)		
Light Source	B	W	Y	B	W	Y	B	W	Y	B	W	Y	B	W	Y	B	W	Y
On state	500			1300			1720			1200			302			419		
Off state	11.8	12	12.4	8.5	9.7	13.5	1.2	1.6	2.2	1.9	1.9	2.0	0.4	0.4	0.4	0.4	0.6	0.7
ACR	84	82	80	154	134	97	1182	877	581	343	344	315	725	750	719	986	733	576
